# Physician-Patient Relationship in Current Cosmetic Surgery Demands More than Mere Respect for Patient Autonomy—Is It Time for the Anti-Paternalistic Model?

**DOI:** 10.3390/medicina58091278

**Published:** 2022-09-14

**Authors:** Mihaela Hostiuc, Sorin Hostiuc, Mugurel Constantin Rusu, Oana-Maria Isailă

**Affiliations:** 1 Department of Internal Medicine, Faculty of Medicine, Carol Davila University of Medicine and Pharmacy, 020021 Bucharest, Romania; 2 Department of Legal Medicine and Bioethics, Faculty of Dental Medicine, Carol Davila University of Medicine and Pharmacy, 020021 Bucharest, Romania; 3 Department of Anatomy, Faculty of Dental Medicine, Carol Davila University of Medicine and Pharmacy, 020021 Bucharest, Romania

**Keywords:** bioethics, surgery, plastic, physicians

## Abstract

The ethical framework of cosmetic surgery is distinct from the one associated with clinical medicine. This distinctiveness has led to significant difficulties in conceptualizing the physician-patient relationship (PPR), as most models have been developed specifically for the latter. The purpose of this article is to show that the PPR in cosmetic surgery can be better described through a distinct approach that we name the anti-paternalistic model of the PPR, and we will briefly present the differences between it and autonomy-based models. We will analyze the principle of non-interference, the variable degree of autonomy of both the patient and the physician within this relationship, the handling of the relevant information, the principle of beneficence as satisfaction, the difficulties regarding the informed consent, the algorithm allowing for the refusal of the procedure, and children-related issues. Based on this analysis, we will show that an anti-paternalistic model of the PPR is preferable to an autonomy-based one, as it allows for better clarification of the underlying ethical issues involved in cosmetic surgery.

## 1. Introduction

The number of aesthetic procedures performed worldwide continues to increase yearly, with the highest number per capita being found in South Korea (898 procedures/100,000 inhabitants), Brazil (669), Colombia (526), and the United States (469) [1]. The main reasons for which patients require cosmetic procedures are pleasure (i.e., to close the gap between their perceived personal body image and the social and cultural standards of beauty) and to resolve psychological issues (such as dysmorphophobia or body dysmorphic syndrome) [2]. The requesting rate is high, but the surgeon must show caution when responding to the patient’s request.

In cosmetic surgery, the medical indication is not indispensable, and therefore, aesthetic procedures may be performed without an objective medical benefit for the patient based on a distinct ethical framework. For example, the principle of beneficence as satisfaction replaces beneficence as a duty to care, quality of life is substituted for the state of optimum physical health as the ultimate goal of medicine, and the risk-to-benefit analysis focuses heavily on non-medical benefits [3,4,5,6,7]. Furthermore, when obtaining informed consent for an aesthetic procedure, physicians have a duty not to interfere with the decision of their patients, especially concerning the recommendation to perform additional or more complex surgical interventions, but with caution and respect for the principle of non-maleficence. When suspicions of psychiatric diagnoses such as BDD [8] or conditions that would endanger the patient’s health or even life (for example increased anesthetic risk, atopic terrain for the materials used, coagulopathies, etc.) have been removed, the anti-paternalistic approach can be brought into the discussion. It can be stated that the anti-paternalistic model appeared after the patient’s request was already being followed in the practice of cosmetic surgery, without a justification based on clinical, objective criteria, but also without a contraindication. Even if psychiatric disorders may stay at the base for recommending a cosmetic intervention in theory, it is not considered a feasible solution, as it does not solve the real, underlying issue [9].

All of these factors have generated ethical, legal, and clinical difficulties in the conceptualization of the PPR in cosmetic surgery, as most models have been developed specifically for clinical medicine [10,11,12,13,14,15]. Most authors working on this particular issue consider a robust autonomy model as best suited for cosmetic surgery; this model has, however, some significant limitations, which we will address below.

In cosmetic surgery, a particular moral approach is needed so that the medical action interferes as little as possible with the patient’s conceptual liberty. The first element in this regard is the awareness of the client/patient boundary, as the person addresses the physician for fulfilling an aesthetic desideratum, without having a somatic pathology [16]. The factors that underlie individual liberty are related to the scale of values of each person. According to John Stuart Mill, “The only freedom which deserves the name is that of pursuing our own good in our own way, so long as we do not attempt to deprive others of theirs, or impede their efforts to obtain it. Each is the proper guardian of his own health, whether bodily, or mental or spiritual. Mankind are greater gainers by suffering each other to live as seems good to themselves, than by compelling each to live as seems good to the rest” [17]. The physician has the duty to respect the liberty of expression of the person giving them the desired aesthetic appearance, without causing harm. Foucault stated that, “We are in the society of the teacher-judge, the doctor-judge, the educator-judge, the ‘social-worker’-judge; it is on them that the universal reign of the normative is based; and each individual, wherever he may find himself, subjects to it his body, his gestures, his behavior, his aptitudes, his achievement” [18]. The physician’s only judgment should be based on medical criteria and the best interest of the patient, even in the form of aesthetic satisfaction. This desideratum, respecting the particular aesthetic standards of the person, could be achieved only through the anti-paternalistic model of the physician-patient relationship.

The purpose of this article is to show that the PPR in cosmetic surgery can be better encompassed through a distinct approach that we name the anti-paternalistic PPR, and we will briefly present the differences between this model and autonomy-based models.

## 2. Materials and Methods

The analysis reported in this article was based on an unsystematized review of the scientific literature in the field. The keywords used for search were “cosmetic surgery”, “plastic surgery”, “autonomy”, “physician-patient relationship”, “anti-paternalism”. This review began with an analysis of the more common models of the PPR, namely those developed by Szasz and Hollender [19], Roter and Hall [20], Emanuel [13], and Ozar [21] (due to the increased similarities between plastic surgery and dental medicine regarding their consumeristic approaches to the therapeutic alliance) as well as models centered on the patient (summarized by Mead and Bower) [22]. We then analyzed the particularities of the therapeutic alliance in plastic surgery, identified the limits of these models, and evaluated whether an anti-paternalistic approach should be preferable.

## 3. Results

### 3.1. Paternalism versus Anti-Paternalism

Paternalism means acting in the best interests of a moral agent without taking into account his/her opinions (or even acting against them) [23]. Paternalism was one of the main pillars of medical ethics until the second half of the 20th century, with beneficence (the duty to aid patients to reach their optimum possible health status) prevailing over autonomy [24,25]. Within this model, the physician has an active role (making most medical decisions). At the same time, the patient is passive because he/she has a decreased interest in assuming responsibility for the medical act and/or does not have the decisional capacity (therefore rendering him/her unable to make medical decisions) [26]. In older paternalistic models, the patient was not even allowed to have an active role based on the adherence to concepts such as the right to treat (prevalent in the past in the European and especially French medical doctrines) [27,28] or the absolute duty to do good [29]. With the rise of the concept of autonomy as the core principle of bioethics [30], models based on autonomy became prevalent [13]. These models saw the patient as an active partner (who should make medical decisions based on the material information received from the physician), while the role of the doctor was seen as more or less passive (even though he/she could recommend a particular course of action or even persuade the patient to act on his/her medical interest) [31]. The medical decision in these models is either taken on by the patient (a pure autonomy model) or is shared, with the patient having the final word, in mixed models (like the interpretative or deliberative models of the PPR) [13].

Anti-paternalism is defined by the view that there should not be any artificially imposed limits upon a person’s self-determination for their own good (limits can, however, be imposed when there are conflicts with the rights of other individuals). This is clearly distinct from autonomy, in which self-determination is dependent upon the presence of a rational will [31].

For example, based on the principle of anti-paternalism, we should not actively interfere with a person using high-risk drugs if other persons are not put at risk. The use of high-risk drugs is contingent upon desire—the consumer does not necessarily want to use the drugs (and thus does not act autonomously, as he/she is under a significant amount of control that is minimizing his/her capacity to act voluntarily) [30]. Rather, the consumer just desires to do so. As John Stuart Mill said, “In the part that says about only himself, his independence is, of right, absolute. About himself, his body and mind, the individual is sovereign” [17]. Gerald Dworkin, in a similar manner, argues that, “Any sensible view has to distinguish between good done to agents at their request or with their consent, and good thrust upon them against their will. So, the normative options seem to be just two. Either we are never permitted to aim at doing good for others against their wishes, and in ways which limit their liberty, or we are permitted to do so” [32]. Even though anti-paternalism seems similar to the principle of autonomy, there are some important distinctions between them (summarized in Table 1 and detailed below).

### 3.2. Anti-Paternalism versus Autonomy

As a general rule, physicians should choose a particular model of the PPR based either on the pathology of the patient [19], or on cues about him/her obtained during the initial interactions [13]. Let us take the following case as an example: an English teacher comes to an emergency room complaining of gastric pain. She is diagnosed with a complicated peptic ulcer, and the right course of action is surgery, but she can also be treated with anti-acid drugs and close surveillance (including blood work to detect hemoglobin levels). When the physician informs her about the optimal course of treatment, she begins to correct the grammatical errors in the surgeon’s speech and fails to discuss anything related to her medical problem. In this case, the cues suggest that the patient does not want a surgical intervention, which was recommended by the physician based on professional guidelines, but is unable to express this wish, as she does not want to be seen as being afraid (teachers are usually in control in front of their students, and fear should not be shown); this fear increases her verbal aggression toward the physician, who should then switch to an interpretative model of PPR specifically to aid the patient in deciding upon what she wants. The physician might tell her, “I know I make English mistakes, and I am sorry. I was on call for the last 24 h, and I did not sleep at all. Surgery is indeed the best medical option, but if you feel this would pose too much of a burden due to your complicated schedule, we could try using proton pump inhibitors for two days with you staying in the hospital; we will take blood samples often, and if your biological parameters stabilize, you will not need surgery after all”. In this case, theoretically, the best model to handle the patient, based strictly on demographic data (highly educated, middle-aged, and the teacher), would be a pure, autonomy-based model [13,20]. However, the increased anxiety of the patient makes this model suboptimal when applied to this particular case, and the physician should switch to an interpretative model [13].

In the autonomy-based models of the PPR, respect for autonomy is requested by the patient, either directly or indirectly, and guarded by society with specific regulations (including laws or codes of ethics). However, within the therapeutic alliance between the patient and the plastic surgeon, this is not necessarily the case. The physician does not have this liberty to evaluate the best model for his/her patient, as most of them are not applicable. Any model in which the physician is in control might significantly increase the risk of malpractice claims, even if the results of the intervention are optimal from a medical point of view (see the discussion below regarding the specific duty of the result). Even a pure autonomous model of interaction with the patient usually entails that the physician check the voluntariness of the patient to make therapeutic recommendations and mandate him/her to take into account the medical benefit (the medical intervention that is chosen by the patient should always have a potential medical benefit) [33].

In aesthetic surgery, there are usually no clear-cut medical indications, and the medical benefit of the patient is therefore in doubt. Many patients seeking plastic surgeons do not necessarily want a purely informative model of the professional relationship—many have psychiatric disorders (cases in which a purely autonomous model is not feasible), and many are in doubt regarding the need for cosmetic surgery or the type of cosmetic procedure. Any type of recommendation, in these cases, can be considered as a conflict of interest [34]. Therefore, if an autonomous model is not always applicable, and if the choice of the model cannot be left in the hands of the physician (with the other models, in which the physician is involved in the decision-making process, requiring a more significant analysis of the beneficence of the procedure compared to the autonomy-based model), we should seek another model that is not prone to these limitations. Within the anti-paternalistic model we propose here, the one requesting full respect of the autonomy of the patient is the physician, who is not protected from legal consequences if the results of the intervention differ from those expected by the patient. This model maximizes the self-determination of the patient, which is no longer limited by beneficence as the duty to care (as is the case in autonomy-based models), but by non-maleficence (physicians should only try not to harm the patient and not necessarily address a patient’s specific medical problem). As non-maleficence replaces beneficence as the primary opposing principle in this professional relationship, the freedom of the patient should be at the highest possible levels, and the physician must assess and attest to it before obtaining the consent for surgery.

The physician should never interfere with the medical reasoning of the patient requesting a cosmetic alteration, not even as a recommendation, except in cases where the wishes of the patient are exaggerated/associated with increased risks. Suggesting additional procedures or proposing a body change that is more significant compared to the initial request of the patient is strictly forbidden, as it breaches two fundamental ethical principles, namely justice and fidelity. Sometimes, patients do not want the cosmetic procedure to reach a social standard of beauty, but rather to meet a personal one, which may be significantly different from societal norms [35]. In some cases, the patient may have a personal reason for wanting to look a particular way. For example, suppose that a patient wants his legs removed because he is an author wanting to write a book about a person without legs, and thus, he wants to know how his character would be feeling. Should the physician perform this procedure? Even though the intervention seems to be beneficial for the patient, it is not a medical benefit. The risks of the surgical procedure are not very high, but the author in this case would remain with a disability for the rest of his life. The informative (autonomy-based) model of PPR should not allow it, as there is no medical benefit for the procedure, and the risks are more than minimal. However, prosthetics nowadays have advanced significantly, and the future disability can be easily managed; moreover, refusing to perform this procedure might be considered discriminatory, as the physician would have refused the procedure based on preconceived notions of normal, from which persons without legs are excluded. In an anti-paternalistic model of the PPR, the physician may perform the procedure—even if there is a lack of medical indication, the procedure can still be performed based on the principle of beneficence as satisfaction (see below), as long as the risks (both medical and non-medical) are manageable.

We could also argue that the driving force behind the request for an aesthetic procedure is desire (the novelist wants to experience how a character from his future novel would feel) and not rational will (rationality imposes limits upon performing disabling procedures without obvious medical indications). In such a case, however, there is no obligation for the physician to do the surgery, as he/she can exit the PPR at any time before performing the intervention based on moral grounds (the conscience clause) [35,36,37] associated with the lack of a medical need mandating an active intervention to restore/improve health. This is, again, different from any other type of PPR, in which, once a medical need has been identified, the physician can only end the professional alliance in certain conditions, such as sending the patient to another, more competent physician for the medical need of the patient, recommending another physician with specific competencies for managing the disease of the patient, recommending another medical institution (one that is better suited for the disease of the patient), or by giving notice to the patient to seek another physician. The latter can be allowed only in non-emergencies if the patient is not discriminated against, and only if there is either a significant lack of understanding between the physician and the patient, the patient is aggressive, or the patient wants a medical intervention that conflicts with the moral/religious beliefs of the physician.

### 3.3. Beneficence and the Anti-Paternalistic Model

Beneficence, seen as a duty to improve the health of the patient, is a moral obligation for any physician. Nevertheless, this is not the case in cosmetic surgery, where non-medical well-being is the driving force for medical interventions, leading to the development of the concept of beneficence as satisfaction. According to this principle, a medical procedure is justifiable from a moral point of view even if it does not have a proper medical indication, as it may lead to benefits for the patients in other dimensions (including social, economic, cultural, psychological, and so on) [38,39,40], and therefore is intrinsically linked with desire. This approach has been accepted as a morally acceptable endpoint of medicine in the most recent revision of the World Medical Association’s Declaration of Geneva, which now states that “The health and **well-being** (emphasis added) of my patient will be my first consideration” [33]. Adding well-being to health as a primary consideration of medical care allows more leniency to physicians in the way they conceptualize the end-goals of care, as quality of life and non-medical aspects of care are becoming more relevant.

### 3.4. The Duty of Result and the Anti-Paternalistic Model

Another distinction resides within the duty of the physician—from a duty of diligence to a duty of result. The duty of diligence (or means) signifies that the physician has the responsibility to aid the patient to the best of his/her knowledge/tools, and if something goes wrong during the treatment (including complications of the intervention or even death), he/she is not liable. The obligation of result means that the physician is bound to obtain the result promised to the patient. For instance, if the result of a mastopexy does not look similar to the one discussed with the patient before the medical intervention and agreed upon, the physician is liable, even if he/she acted diligently [41,42]. Informative models of PPR are based on the duty of diligence—the patient chooses a procedure, and the physician should, to the best of his/her knowledge, try to maximize the medical benefit for the patient within the confinements of the obtained consent, which was ideally obtained after a proper exchange of relevant information. If the physician has a duty of result, the informative models are not applicable; the given information, even though it must be relevant for the patient, is not enough to limit the liability of the physician if the desired result is not reached. Again, desire is the driving force beneath the request to have the procedure performed and not a medical need. Thus, such an approach is, by definition, anti-paternalistic.

In the anti-paternalistic model, informed consent should be based, to a more substantial degree compared to other models, on a lack of control on the part of the physician. Any type of external interference from the physician directed toward recommending a particular cosmetic intervention or encouraging the performance of a different procedure to further “improve” the looks of the patient is strictly forbidden. Unlike in the autonomy model of PPR, where the physician must present all relevant information to the patient to allow for an informed decision, in this anti-paternalistic model, the information should be more exhaustive. It should also include information about all potential, known risks of the intervention (even if not deemed relevant), non-medical consequences (e.g., if the alterations requested are outside social norms, they may cause an increased risk of social exclusion or decreased job opportunities), the probability of not reaching the desired result, the worst possible outcomes of the interventions, the expected costs of the procedure, and also the costs of treating the most frequent complications. This extensive information is needed because, sometimes, a misdirected desire can be overcome by rationality provided that the information reaches a soft spot with the patient. It is especially relevant when the request of the patient is directed toward an intervention outside recognized social norms.

### 3.5. Conscience Clause and the Anti-Paternalistic Model

Some physicians refuse cosmetic procedures outside social norms due to conflicts with their moral values (conscientious objection), which are usually caused by one of the following: (1) the aims of the treatment are outside the traditional purposes of medicine and outside standardized cosmetic interventions; (2) the motivation to undergo the intervention is trivial, making the risks unacceptable; (3) the intervention is aimed at promoting cultural, religious, or political views with which the physician disagrees; or (4) the procedure would generate some benefit for the patient, but might be detrimental for the society at large [35]. Even if the latter are difficult to be judged by a physician—e.g., how can a physician establish which procedure may be accepted or refused based on political conscience clauses?—we must emphasize that they are the ones who should decide when to activate this clause, which can be done either based on personal (moral/ethical) reasons or on the perceived effects of that procedure on society. In the anti-paternalistic model, physicians should have an increased liberty to access the conscience clause, as a counterbalance to the increased liberties allocated to the patients.

Refusal of these procedures can also be justified by using the dichotomy between negative and positive autonomy. Negative autonomy means that the patient has the right to refuse a procedure that is clearly in his/her benefit, even if this refusal may cause death. Positive autonomy means that the patient has the right to request a medical procedure irrespective of its medical consequences [7], but the physician has the right (or even the obligation in particular cases) to refuse to perform it. Physicians have an absolute duty to respect negative autonomy (and therefore the informed refusal of the procedure), while the positive autonomy of the patient is relative and should always be counterbalanced by the principles of non-maleficence (doing no harm to the patient through an active intervention) and professional independence (which is highly dependent upon a medical indication for the requested procedure).

Non-maleficence means doing no harm to the patient through active medical practices. This principle should be evaluated whenever there are potential negative consequences of the procedure and should always be evaluated conjointly with beneficence. It should be noted that the risks associated with a medical intervention, which have a medical indication, and which are accepted by the patient, are not considered maleficent. In cosmetic surgery, non-maleficence is a weaker concept in the literature, being found in arguments justifying procedures that might be harmful to the patient, as they may lead to other, non-medical benefits [35]. For example, there is the case of Dennis Avner, who had 14 surgical procedures aimed at increasing his resemblance to a tigress (including extensive tattooing, facial subdermal implants, nasal septum relocation, silicone injections, bifurcation of the upper lip, filling and capping of his teeth, elongation of the earlobes, change in hairline location, and transdermal head implants) [43,44]. These procedures obviously contradict the classical concept of non-maleficence, as it is difficult to justify putting a patient at the risk of multiple surgeries to generate a look significantly outside social norms. In other words, these procedures may cause significant non-medical harm along with increased difficulties of social and economic integration. These alterations may also cause psychiatric disorders if they were not already present (in this case, they may have been underlying this need to appear as a tigress, as the patient ultimately committed suicide, confirming that the result of these procedures was not beneficent for him).

The reasoning for weakening the concept of non-maleficence in cosmetic surgery is the principle of justice. Little, for example, has argued that cosmetic interventions not complying with social norms might be grounded in injustice perpetrated against specific groups of people. For example, if a non-Caucasian person wants an intervention to look more Caucasian, the underlying reasoning might not be an actual dissatisfaction with the way he/she looks, but rather based on a fear of being a victim of racism [45]. As presented above, this weakened principle of non-maleficence should be counterbalanced with the self-determination of the patient in the anti-paternalistic model, therefore allowing for a procedure without a medical indication to be performed, provided that the risks are not significant for the patient.

Furthermore, from a social and a medical-legal standpoint, in cosmetic surgery we can talk about the obligation of the result, which depends on the patient’s perception but also on the extrinsic factors (when they exist and are of great importance for the patient) that led to the decision to request the intervention. There is a risk that the social result will not be the one expected by the patient, which the physician must consider, that is to know and understand the patient’s scale of values before intervening.

If, however, the risks are significant for the patient, as assessed by the physician, the anti-paternalistic model should not allow the procedure, based on the conjoint force of three concepts, non-maleficence, conscience clause, and duty of result, whose relative importance for each case should be judged by the physician. A procedure may be refused based on a single concept—e.g., in Dennis Avner’s case, non-maleficence or the conscience clause should be enough, in our opinion, to refuse it. Other procedures could be refused based on an association of concepts—for example, a whitening procedure could be refused based on both non-maleficence and the conscience clause, while if the patient was also diagnosed with body dysmorphic disorder, the duty of result should also be taken into consideration.

One might argue that the extensive use of the conscience clause may sometimes interfere with the right to treatment. For example, suppose that a patient with an autoimmune disorder approaches a cosmetic surgeon for breast implantation. After extensive discussions, the physician refuses to perform the procedure due to increased medical risks, arguing that increased risks are not justifiable for the perceived benefit. In this case, there is an actual conflict between the physician’s conscience and the patient’s wishes, not the right to treatment, as the intervention is cosmetic and not aimed at solving a medical disorder. The physician should not interfere with the medical reasoning of the patient (they should not, for example, recommend an alternative procedure or convince the patient that another type of implant is better) but rather only refuse to perform it.

### 3.6. Patients with Decreased Decisional Capacity and the Anti-Paternalistic Model

In clinical medicine, obtaining informed consent for any medical procedure that has more than minimal risks is mandatory, but there are exceptions (e.g., an unconscious patient without relatives, patients without decisional capacity such as minors, and so on). Even in autonomy-based models of PPR, the patient can delegate a third party to be informed and make decisions on his/her behalf (respecting the autonomy of the patient also means respecting his/her wish to delegate the responsibility). In the anti-paternalistic model, this is not possible—the intervention does not represent an emergency, and a legal guardian/tutor should not be allowed to make decisions altering the aesthetics of the patient [46].

Autonomy-based models of PPR usually restrict cosmetic interventions on minors. The main reason for this interdiction on moral grounds is the fact that the risks associated with cosmetic procedures are not adequately counterbalanced by the autonomy of the patient (which is mediated through the legal guardians and is therefore significantly less powerful from both a legal and a moral standpoint), and also because there are intrinsic limits to conceptualizing the telos in younger years, with children being significantly more prone to changing their minds in short periods of time. Based on this change of perspective, a young person can easily adopt a different view about a particular look. Some have even argued that these procedures should be criminalized if they fulfill some conditions (i.e., if they are unnecessary, harmful, or non-therapeutic) [47]. However, in recent years, there seems to have been an increased acceptance of aesthetic procedures on children [47,48,49]. In the anti-paternalistic model, they may be allowed, as rationality can be overcome by desire. However, this is only true for emancipated minors who can legally give their consent for the procedure. Parents cannot rationally support a decision based on the desires of their children, as these cosmetic procedures are potentially maleficent, and there is no medical benefit (they can only consent for medical procedures directed toward making their children better from a medical point of view).

In the autonomy model of PPR, physicians may refuse to perform an intervention with no medical indication or if there are not enough resources available to optimize medical care (e.g., a lack of necessary skills or tools). In the anti-paternalistic model, these reasons are not valid—the medical indication is not indispensable, and therefore, aesthetic procedures may be performed without an objective medical benefit for the patient. However, the lack of needed skills or resources should forbid the intervention from taking place, as there is no medical emergency to justify performing a cosmetic procedure in subpar conditions. The physician can refuse the intervention based solely on moral grounds, or if he/she suspects that the patient has other disorders, either non-psychiatric, which would unjustifiably increase the risk of the cosmetic intervention, or psychiatric, which should lead to a recommendation for psychiatric evaluation before surgery [4,50].

Pellegrino has stated that, “The ends of medicine are ultimately the restoration or improvement of health and, more proximately, to heal, that is, to cure illness and disease or, when this is not possible, to care for and help the patient to live with residual pain, discomfort, or disability” [51]. Even if this approach is currently disputed, it summarizes what most medical ethicists in the past, and what many medical ethicists today, consider to be the defining goals of medical care. Medical interventions not fulfilling these goals often require additional ethical safeguards for their justification. For example, medical research ethics is currently a separate subdiscipline of bioethics. Many fundamental principles, even if they sound like those of medical ethics, are analyzed distinctly, and their meaning is significantly different. Similarly, cosmetic surgery has a specific set of obligations and principles that must be respected, in contrast with clinical medicine and which have been summarized in this article, within this new model of the physician-patient relationship.

Most of these additional principles and constructs (such as the concept of beneficence as satisfaction) are imposed on physicians who perform cosmetic surgery. However, it is the patient who seeks the procedure (as the physician does not have the duty to pursue the maximum degree of medical benefit), allowing him/her to exert almost full control on the professional relationship. This is different from the case of non-aesthetic medicine, in which the control is either shared (autonomy-based PPRs) or in the hands of the physician (paternalistic PPRs). Therefore, even if this sounds demeaning for the physician, this decreased control makes him/her an executant, and the relationship with the patient is closer to a civil, consumerist contract than a medical, professional relationship. It is not, however, a purely merchant-consumer contractual relationship, as the physician still has specific duties derived from the fact that medicine is a profession and not a business [21]. A profession is defined by a series of characteristics regarding prestige, innate and acquired abilities, know-how, protected markets, control, identity, shared values, moral aspects, interprofessional relations, and so on [52].

The cosmetic surgeon should perform the requested procedure in such a manner as to reach the required aesthetic desideratum (for which the surgeon is competent due to specific training) and to minimize the potential harm done to the patient, both that caused by the medical intervention per se (postoperative complications) and by the actual result (if it is not close to societal aesthetic norms). The surgeon can always refuse to perform the procedure (having full autonomy within the relationship with the patient) but should never actively recommend an augmentative medical procedure leading to a more significant departure from the baseline compared to what the patient initially wanted.

**Figure 1:**
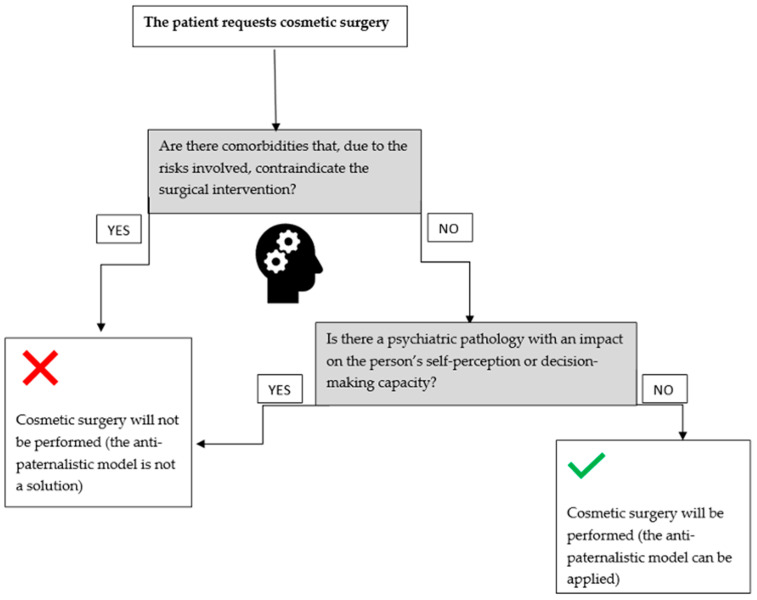
The algorithm for the anti-paternalistic model applicability when a patient requests cosmetic surgery [4,8].

The aforementioned aspects can be represented by the following algorithm (Figure 1).

The main limits of this model may include cases in which the cosmetic procedure is requested as an addition to a therapeutic procedure (e.g., cosmetic interventions after burns), patients with significant psychiatric disorders (e.g., body dysmorphic disorder), in which the cosmetic procedure is requested as a consequence of an underlying psychiatric pathology (for details see [8]), or legislative regulations interfering with this model’s approach (e.g., restrictive regulations regarding the conscience clause, which would render this model inapplicable as there would not be a significant counterbalance to the strong autonomy of the patient within the therapeutic alliance).

## 4. Conclusions

The use of an anti-paternalistic model of the PPR allows for clarification of the intrinsic relationship between these two contractual parties from an ethical point of view and establishes which ethical principles should be prioritized while performing aesthetic procedures. It is better suited for the particularities of the cosmetic surgery profession, which has not been extensively evaluated from this point of view until now. Additionally, the model allows for a more precise delineation of aesthetic surgery as a profession and not a business, which should increase the trust in these physicians, who are to be seen primarily as physicians and not as sellers of medical products.

## Figures and Tables

**Table 1:** Differences between autonomy and anti-paternalist models of PPR.

Principle	Autonomy-Based PPR	Anti-Paternalism-Based PPR
Basis for self determination	Rationality	Rationality and desire
The person imposing non-interference with medical decisions	Patient	Physician
Non-interference with medical decisions by the future patient	Strong	Absolute
Counterbalance from the duty to care	Strong	Weak
Respecting non-maleficence	Strong	Weak
Beneficence	Based on duty to care	Based on beneficence as satisfaction
Quantity of information disclosed to the patient	Material	Exhaustive
The basis of the contractual relationship between physician and patient	Duty to care (duty of diligence)	Duty of result
Minors	Mediated autonomy (through legal representatives)	Non-mediated (should not be allowed, unless emancipated minors)
Circumvention of informed consent	Possible (emergencies, lack of decisional capacity)	Not possible
Refusal from physicians	Lack of medical indication Lack of resources	Additional medical disorders Conscience clause

## References

[1] Heidekrueger P.I., Juran S., Ehrl D., Aung T., Tanna N., Broer P.N. (2017). Global aesthetic surgery statistics: A closer look. J. Plast. Surg. Hand Surg..

[2] Atiyeh B.S., Rubeiz M.T., Hayek S.N. (2008). Aesthetic/cosmetic surgery and ethical challenges. Aesthetic Plast. Surg..

[3] Alsarraf R. (2000). Outcomes research in facial plastic surgery: A review and new directions. Aesthetic Plast. Surg..

[4] Margraf J., Meyer A.H., Lavallee K.L. (2013). Well-being from the knife? Psychological effects of aesthetic surgery. Clin. Psychol. Sci..

[5] Badiu D., Nastasel V. (2018). Reproductive Technologies Used by Same Gender Couples. Clinical Ethics at the Crossroads of Genetic and Reproductive Technologies.

[6] Brazier M., Lobjoit M. (2005). Protecting the Vulnerable: Autonomy and Consent in Health Care.

[7] Saad T.C. (2018). Mistakes and missed opportunities regarding cosmetic surgery and conscientious objection. J. Med. Ethics.

[8] Hostiuc S., Isailă O.-M., Rusu M.C., Negoi I. (2022). Ethical Challenges Regarding Cosmetic Surgery in Patients with Body Dysmorphic Disorder. Healthcare.

[9] Vázquez-Bourgon J., Ruiz E.G., Zatón F.H., Carulla L.S., Arriola R.A., Gutiérrez D.T., Facorro B.C. (2021). Differences between psychiatric disorders in the clinical and functional effectiveness of an acute psychiatric day hospital, for acutely ill psychiatric patients. Rev. Psiquiatr. Salud Ment..

[10] Borza L.R., Gavrilovici C., Stockman R. (2015). Ethical Models of Physician--Patient Relationship Revisited with Regard to Patient Autonomy, Values and Patient Education. Med.-Surg. J..

[11] Borrel-Carrio F., Suchman A., Epstein R. (2004). The Biopsychosocial Model 25 Years Later: Principles, Practice, and Scientifi c Inquiry. Ann. Fam. Med..

[12] Thomasma D.C. (1983). Beyond medical paternalism and patient autonomy: A model of physician conscience for the physician-patient relationship. Ann. Intern. Med..

[13] Emanuel E.J., Emanuel L.L. (1992). Four models of the physician-patient relationship. JAMA.

[14] Ozar D.T. (1984). Patients’ autonomy: Three models of the professional-lay relationship in medicine. Theor. Med..

[15] Hostiuc S., Buda O., Mihailov C.I., Hostiuc M. (2015). New technologies in biomedicine: Opinions of young Romanian physicians. Acta Bioeth..

[16] Nejadsarvari N., Ebrahimi A., Ebrahimi A., Hashem-Zade H. (2016). Medical Ethics in Plastic Surgery: A Mini Review. World J. Plast. Surg..

[17] Mill J.S. (2002). The Basic Writings of John Stuart Mill: On Liberty, the Subjection of Women, and Utilitarianism.

[18] Foucault M. (1995). Discipline & Punish: The Birth of the Prison.

[19] Szasz T., Hollender M. (1956). A Contribution to the Philosophy of medicine: The Basic Models of the Doctor-Patient Relationship. AMA Arch. Intern. Med..

[20] Roter D.L., Hall J.A. (2006). Doctors Talking with Patients/Patients Talking with Doctors.

[21] Ozar D.T., Sokol D.J., Patthoff D.E. (2018). Dental Ethics at Chairside.

[22] Mead N., Bower P. (2000). Patient-centredness: A conceptual framework and review of the empirical literature. Soc. Sci. Med..

[23] Sullivan L.S. (2016). Medical maternalism: Beyond paternalism and antipaternalism. J. Med. Ethics.

[24] Hostiuc S., Buda O. (2018). The Age of Informed Consent: A European History.

[25] Hostiuc S., Buda O. (2013). Physician-Patient Relationship Before the Second Half of the XIXth Century. Proc. Rom. Acad. Ser. B.

[26] Roter D.L. (1977). Patient participation in the patient-provider interaction: The effects of patient question asking on the quality of interaction, satisfaction and compliance. Health Educ. Monogr..

[27] Minovici N., Stănescu I. (1939). Etica Responsabilității Medicale. Rev. Med. Leg..

[28] Zdanowicz M.T. (2008). Refusing the Right to Refuse: Coerced Treatment of Mentally Disordered Persons. Psychiatr. Serv..

[29] Percival T. (2014). Medical Ethics.

[30] Beauchamp T.L., Childress J.F. (2001). Principles of Biomedical Ethics.

[31] Kant I. (1949). Critique of Practical Reason, and Other Writings in Moral Philosophy.

[32] Dworkin G., Zalta E.N. Paternalism. Stanford Encyclopedia of Philosophy.

[33] The World Medical Association (2015). WMA Declaration of Geneva. Int. J. Pers. Cent. Med..

[34] Swanson E., Brown T. (2018). A Discussion of Conflicts of Interest in Plastic Surgery and Possible Remedies. Plast. Reconstr. Surg. Glob. Open.

[35] Minerva F. (2017). Cosmetic surgery and conscientious objection. J. Med. Ethics.

[36] Pope T.M. (2010). Legal briefing: Conscience clauses and conscientious refusal. J. Clin. Ethics.

[37] Hostiuc S., Badiu D., Năstășel V., Hangan T.L., Marinescu M. (2017). Conscience clause and its potential applicability in reproductive medicine. Gineco.eu.

[38] Press I. (2007). Patient Satisfaction Understanding and Managing the Expen ence of Care. Health Prog..

[39] De Mello E.S.C. (1994). C-sections as ideal births: The cultural constructions of beneficence and patients’ rights in Brazil. Camb. Q. Healthc. Ethics.

[40] Crerand C.E., Magee L. (2013). Cosmetic and reconstructive breast surgery in adolescents: Psychological, ethical, and legal considerations. Seminars in Plastic Surgery.

[41] Di Lorenzo P., Casella C., Capassso E., Conti A., Fedeli P., Policino F., Niola M. (2018). The central importance of information in cosmetic surgery and treatments. Open Med..

[42] Piras M., Delbon P., Conti A., Graziano V., Capassso E., Niola M., Bin P. (2016). Cosmetic surgery: Medicolegal considerations. Open Med..

[43] Parry L. Stalking Cat Confirmed Dead at 54.

[44] Casavant V.R. Catman’s Transformation Raises Concerns over Extreme Surgery. The Seattle Times.

[45] Little M.O. (1998). Cosmetic surgery, suspect norms, and the ethics of complicity. Enhancing Human Traits: Ethical and Social Implications.

[46] Hill C. (2007). Anti-Anti-Anti-Paternalism.

[47] Baker D.J. (2014). Should Unnecessary Harmful Nontherapeutic Cosmetic Surgery be Criminalized?. New Crim. Law Rev..

[48] Larson K., Gosain A.K. (2012). Cosmetic surgery in the adolescent patient. Plast. Reconstr. Surg..

[49] Berer M. (2010). Cosmetic Surgery, Body Image and Sexuality. Reprod. Health. Matters..

[50] Sarwer D.B., Spitzer J.C. (2012). Body image dysmorphic disorder in persons who undergo aesthetic medical treatments. Aesthetic Surg. J..

[51] Pellegrino E.D. (1990). The Medical Profession as a Moral Community. Bull. N. Y. Acad. Med..

[52] Goode W.J. (1957). Community within a community: The professions. Am. Sociol. Rev..

